# Bacterial Microbiota Associated with the Glacier Ice Worm Is Dominated by Both Worm-Specific and Glacier-Derived Facultative Lineages

**DOI:** 10.1264/jsme2.ME16158

**Published:** 2017-03-09

**Authors:** Takumi Murakami, Takahiro Segawa, Roman Dial, Nozomu Takeuchi, Shiro Kohshima, Yuichi Hongoh

**Affiliations:** 1 Department of Biological Sciences, Tokyo Institute of TechnologyTokyo 152–8550Japan; 2 Center for Life Science Research, University of YamanashiYamanashi 409–3898Japan; 3 National Institute of Polar ResearchTokyo 190–8518Japan; 4 Transdisciplinary Research Integration CenterTokyo 105–0001Japan; 5 Department of Environmental Science, Alaska Pacific UniversityAlaska 99508USA; 6 Department of Earth Sciences, Chiba UniversityChiba 263–8522Japan; 7 Wildlife Research Center, Kyoto UniversityKyoto 606–8203Japan

**Keywords:** enchytraeid, glacier ecosystem, gut bacteria, symbiosis, psychrophilic

## Abstract

The community structure of bacteria associated with the glacier ice worm *Mesenchytraeus solifugus* was analyzed by amplicon sequencing of 16S rRNA genes and their transcripts. Ice worms were collected from two distinct glaciers in Alaska, Harding Icefield and Byron Glacier, and glacier surfaces were also sampled for comparison. Marked differences were observed in bacterial community structures between the ice worm and glacier surface samples. Several bacterial phylotypes were detected almost exclusively in the ice worms, and these bacteria were phylogenetically affiliated with either animal-associated lineages or, interestingly, clades mostly consisting of glacier-indigenous species. The former included bacteria that belong to *Mollicutes*, *Chlamydiae*, *Rickettsiales*, and *Lachnospiraceae*, while the latter included *Arcicella* and *Herminiimonas* phylotypes. Among these bacteria enriched in ice worm samples, *Mollicutes*, *Arcicella*, and *Herminiimonas* phylotypes were abundantly and consistently detected in the ice worm samples; these phylotypes constituted the core microbiota associated with the ice worm. A fluorescence *in situ* hybridization analysis showed that *Arcicella* cells specifically colonized the epidermis of the ice worms. Other bacterial phylotypes detected in the ice worm samples were also abundantly recovered from the respective habitat glaciers; these bacteria may be food for ice worms to digest or temporary residents. Nevertheless, some were overrepresented in the ice worm RNA samples; they may also function as facultative gut bacteria. Our results indicate that the community structure of bacteria associated with ice worms is distinct from that in the associated glacier and includes worm-specific and facultative, glacier-indigenous lineages.

Glaciers, which are harsh environments that are permanently covered with ice and snow, have unique ecosystems composed of psychrophilic/psychrotolerant organisms (3, 33). Recent studies revealed that microorganisms thriving on glacial surfaces have a marked impact on global biogeochemical cycles and surface ice melting (2, 34, 37, 40). Although the importance of glacier ecosystems has been recognized and many glaciers have been examined, most studies have focused on microorganisms on the glacier surface, and knowledge on glacier invertebrates is limited.

The glacier ice worm, *Mesenchytraeus solifugus* (phylum Annelida; family Enchytraeidae), is the largest metazoan and regarded as the predominant consumer in North American maritime glaciers (17, 35). Ice worms are nocturnal in summer seasons; they reside deep beneath the glacier surface in the daytime to avoid sunlight, and emerge at dusk on the surface to forage food, which mainly consists of unicellular green algae (17, 35). Their behaviors in winter seasons are unknown. Their physiology and phylogeny have also been investigated (9, 14, 18, 38); however, limited information is available on their ecological roles in glacial environments. In our previous study, the bacterial community structures physically associated with the ice worm were analyzed by cloning bacterial 16S rRNA genes (27). The findings obtained revealed that the ice worm harbors glacier-derived and animal gut-specific bacterial lineages, and that the novel *Mollicutes* bacterium, “*Candidatus* Vermiplasma glacialis,” colonizes the gut wall of ice worms as a dominant bacterial member. However, the number of analyzed specimens and sequences were limited; therefore, more comprehensive surveys are needed in order to obtain a deeper understanding of the community structure of bacteria associated with ice worms.

In the present study, we aimed to clarify the taxonomic composition of the bacterial microbiota associated with the ice worm in more detail by deep sequencing 16S rRNA gene amplicons. In order to identify the core symbiotic microbiota, if one exists, ice worms were collected from two distinct glaciers in Alaska, Harding Icefield and Byron Glacier, and their associated bacterial communities were compared. The 16S rRNA gene and its transcripts were simultaneously analyzed to assess the activity of each bacterial species. Furthermore, bacterial community structures from the glacier surfaces were examined, and the specificity of the bacterial species associated with ice worms was evaluated. Our results provide an insight into the unique niche occupied by ice worms with their associated bacterial consortium in glacial ecosystems.

## Materials and Methods

### Sample collection

Sampling expeditions were carried out in the upper area of Exit Glacier located in Harding Icefield (August 2014) and in Byron Glacier (August 2015). Both glaciers are located in the Kenai Peninsula, Alaska, and are approximately 90 km apart. In Harding Icefield, sampling was conducted at two sites; an ice surface site (60°09.622′N, 149°47.482′W) and snowpack site (60°09.167′N, 149°46.795′W) (Table S1). In Byron Glacier, samples were collected from the ice surfaces of an avalanche cone (60°45.672′N, 148°50.89′W) below the terminus of the glacier. Ice worms and glacier surface samples were collected with sterile stainless steel scoops. All ice worm samples were collected at dusk when ice worms started to appear on the surface of the glaciers. Specimens were preserved on site in RNA*later*^®^ solution (Ambion, Austin, TX, USA). Some collected ice worms were kept alive in melted glacier surface ice. All fixed samples were kept frozen with ice during transportation to a laboratory in Japan. Specimens preserved in RNA*later*^®^ were stored at −80°C, and live ice worms were reared at 4°C.

### Nucleic acid extraction

All ice worm and glacier ice specimens in RNA*later*^®^ solution were slowly thawed on ice. Five ice worm individuals were arbitrarily selected from the specimens and placed into one tube. DNA and RNA were both simultaneously extracted using the PowerViral^®^ Environmental RNA/DNA Isolation Kit (MO BIO Laboratory, Carlsbad, CA, USA), according to the manufacturer’s protocol with modifications. Briefly, ice worms were suspended in 600 μL of solution PV1 (lysis buffer), in which 24 μL of 1M dithiothreitol (DTT) was added instead of 6 μL β-mercaptoethanol. The suspension was homogenized with a sterile plastic pestle and subjected to a bead-beating procedure. Two-milliliter aliquots from glacier ice samples were centrifuged at 10,000×*g* at 4°C for 20 min. The pellets were suspended in solution PV1 with DTT, as described above, and processed according to the manufacturer’s protocol. Residual RNA or DNA was digested using RNaseA (Wako, Osaka, Japan) or the TURBO DNA-free™ Kit (Ambion), respectively. DNA extracts were further purified with the DNeasy^®^ Blood and Tissue Kit (Qiagen, Hilden, Germany) to remove RNaseA. The amplification of 16S rRNA genes was not observed from purified RNA samples in 30-cycle PCR with a *Bacteria*-specific primer set, performed as described previously (27).

### PCR, RT-PCR, and amplicon sequencing

RNA samples were reverse-transcribed into cDNA using the SuperScript^®^ III First-Strand Synthesis System (Invitrogen, Carlsbad, CA, USA) with random hexamers. PCR was conducted using Phusion^®^ High-Fidelity DNA Polymerase (New England Biolabs, Ipswich, MA, USA) with the *Bacteria*-specific 341F and 785R primer set, which targets the V3–V4 region (approx. 400 bp) of 16S rRNA. Primer sequences contained the Illumina overhang adapter sequence (http://support.illumina.com/downloads/16s_metagenomic_sequencing_library_preparation.html). The PCR program was as follows: initial denaturation at 98°C for 30 s, 25 cycles of denaturation at 98°C for 10 s, annealing at 55°C for 30 s, extension at 72°C for 45 s, and a final extension at 72°C for 7 min. Nextera^®^ XT indices (Illumina, San Diego, CA, USA) were attached to the amplicons by eight additional PCR cycles. After purification with the Agencourt^®^ AMPure^®^ XP (Beckman Coulter, Brea, CA, USA), DNA was quantified with the Qubit^®^ dsDNA High Sensitivity Assay Kit (Invitrogen). Sequencing was conducted on the Illumina MiSeq™ platform with MiSeq reagent kit v3.

### Quality check of sequence reads and assignment to operational taxonomic units (OTUs)

Paired-end sequence reads were merged, primer trimmed, and quality filtered using the program package USEARCH v8.0 (12). Quality filtering was performed using the *fastq filter* command with the following parameters: minimum length=350 bp, no ambiguous bases allowed, and maximum expected errors for all bases in a read were less than 1. The quality of reads was evaluated using FastQC (http://www.bioinformatics.babraham.ac.uk/projects/fastqc/).

16S rRNA sequences were sorted into OTUs with a criterion of 97% sequence identity using the program UCLUST implemented in the QIIME v1.8.0 package with the furthest-neighbor clustering algorithm (6, 12). Chimeric sequences were identified using UCHIME (13), and singletons were also eliminated from subsequent analyses. The taxonomic assignment of OTUs was performed with the taxonomic classification service implemented in the SINA Alignment Service (https://www.arb-silva.de/aligner/) using the SILVA 126 database with default parameter settings, except for the minimum identity with the query sequence, set to 0.80. OTUs assigned to *Eukarya*, *Archaea*, mitochondria, chloroplasts, and “unclassified” were removed. We also removed OTUs affiliated with the genera *Halomonas* and *Shewanella*; we concluded that these two bacterial groups were reagent contaminants because they commonly appeared in distinct samples, including those not related to this study (data not shown). We further eliminated OTUs that could not be aligned with other 16S rRNA sequences.

### Statistical analysis

The similarity of bacterial community structures was evaluated with QIIME using nonmetric multidimensional scaling (NMDS) based on the Bray–Curtis similarity index. Differences among clusters were statistically tested by an analysis of similarity (ANOSIM) with 999 permutations. The separation of clusters in the NMDS plot was correlated to OTU abundance by calculating Spearman’s correlation coefficient with *mothur* v1.37.0 (29).

In each of the 16S rRNA OTUs recovered from the ice worm samples, differences in abundance between DNA and RNA samples were statistically tested in *R* v3.2.2 using the DESeq2 package with the negative binomial Wald test (23). If the adjusted *P* value was less than 0.05, we considered the OTU abundance to be significantly different between DNA and RNA samples.

### Phylogenetic analysis

Nearly full-length 16S rRNA gene sequences obtained in our previous study (27) were aligned using ARB (24) with manual corrections. In total, 1,312 (for *Arcicella*) and 1,218 (for *Herminiimonas*) unambiguously aligned nucleotide sites, which corresponded to positions 69–1440 and 94–1390 in *Escherichia coli* (J01695), respectively, were used for a phylogenetic analysis. Maximum likelihood (ML) and neighbor-joining (NJ) trees were constructed in MEGA7 (22) with 500 bootstrap resamplings. The GTR+Γ+I nucleotide substitution model and maximum composite likelihood model were used for ML and NJ tree reconstructions, respectively.

### Fluorescence *in situ* hybridization (FISH)

In order to detect *Arcicella* cells associated with ice worms, an *Arcicella*-specific probe, Arci_1251 (5′-GTGTTACCACATAGCG ACCTGC-3′), which corresponds to positions 1251–1272 in *E. coli* (J01695), was designed using ARB and labeled with Texas Red at the 5′ end. A non-labeled helper probe (5′-GGTTTTGTAGATT GGCACT-3′) was used to improve the efficiency of hybridization (16). The preparation of 10-μm-thick cryosections of ice worms was conducted using a Leica CM 1850 cryostat (Leica Biosystems, Nußloch, Germany). Hybridization was performed at 55°C for 2 h, and specimens were enclosed using the SlowFade^®^ Gold Antifade Mountant with 4,6-diamidino-2-phenylindole (DAPI) (Thermo Fisher Scientific, Waltham, MA, USA). Specimens were observed under an Olympus BX51 epifluorescence microscope (Olympus, Tokyo, Japan). Procedures were described in detail previously (27).

### Nucleotide sequence accession numbers

Datasets of 16S rRNA reads sequenced in this study have been deposited in DDBJ/EMBL/GenBank under the accession number DRA005157.

## Results

### Taxonomic composition and species richness of bacteria

Three and six sets of the ice worm samples, each set containing five individual worms, from Harding and Byron (designated as Harding worm/Byron worm), respectively, were subjected to 16S rRNA amplicon sequencing. In addition, two and three sets of the glacier surface samples from Harding and Byron (designated as Harding surface/Byron surface), respectively, were similarly analyzed. In total, 4,208 OTUs were identified from 1,965,105 paired-end reads. The mean number (with SD) of the observed OTUs in each sample category was as follows; 79±16 (Harding worm DNA), 546±350 (Harding worm RNA), 101±44 (Byron worm DNA), 1,105±583 (Byron worm RNA), 230±160 (Harding surface DNA), 757±297 (Harding surface RNA), 116±30 (Byron surface DNA), and 810±274 (Byron surface RNA). Thus, the observed number of OTUs as well as the Chao1 species richness estimate and reciprocal Simpson’s diversity index were higher in RNA samples than in DNA samples in any of the worm or glacier surface samples (Table S1). The rank abundance curves of the OTUs showed that RNA samples contained much rare OTUs than DNA samples (Fig. S1).

**Fig. 1:**
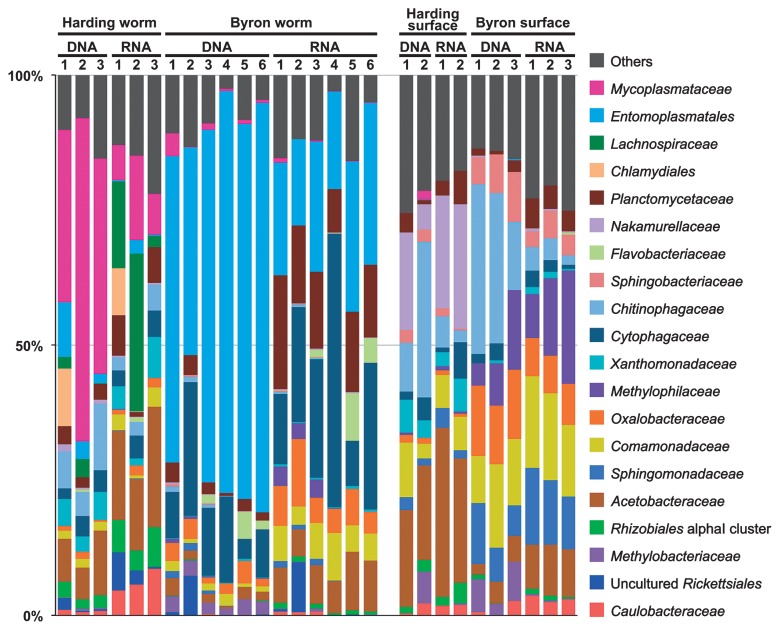
Taxonomic composition of bacteria based on 16S rRNA sequences. This classification was mainly performed at the family level.

The taxonomic composition of bacteria was basically consistent among the biological replicates for each sample category (Fig. 1). In contrast, marked differences were observed among the different sample categories. For example, *Entomoplasmatales* and *Mycoplasmataceae* sequences were abundantly detected in ice worm samples, whereas only a few were recovered from the glacier surface samples. Most of the *Mycoplasmataceae* OTUs in the ice worm samples were identical or closely related to the “*Ca.* Vermiplasma” sequences obtained in our previous study (27), and differed from OTU13756, which accounted for 1.6% of sequences in the “Harding surface DNA 2” sample (Fig. 1). The OTU13756 sequence was identical to the sequences of two *Mycoplasmataceae* bacteria in the NCBI nr/nt database: the hemotrophic cattle pathogen “*Candidatus* Mycoplasma haemobos” (KJ883514) (19) and uncultured bacterium clone GYs3-84 (JX493242) obtained from soil in China (44). *Entomoplasmatales* OTUs were also identical or closely related to the phylotypes obtained in our previous study (27). *Lachnospiraceae* OTUs were exclusively recovered from the Harding worm samples. The closest relative of the dominant *Lachnospiraceae* OTU742 was a sequence obtained from a termite gut sample (AB089001) with 93.3% sequence similarity. *Cytophagaceae*, predominated by the genus *Arcicella* (25 out of 85 *Cytophagaceae* OTUs and 91.8% of the total *Cytophagaceae* reads), were frequently detected in both worm and glacier surface samples, but were more abundant in the former, particularly in Byron worms (Fig. 1).

### Similarity of bacterial community structures

**Fig. 2:**
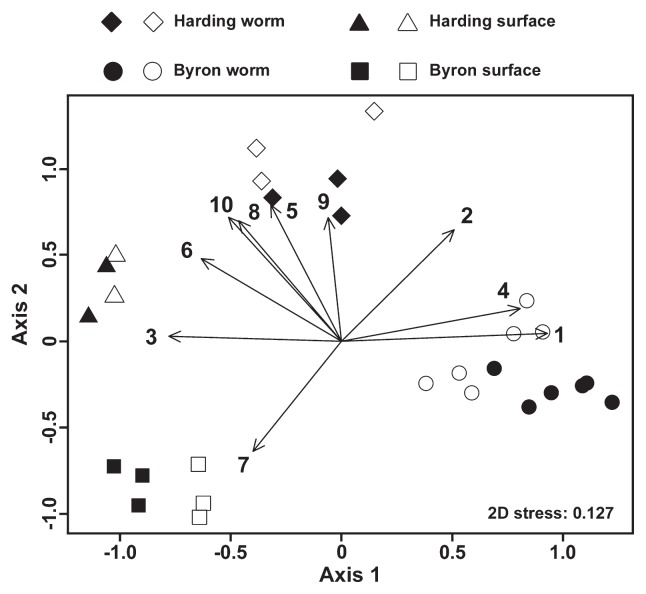
NMDS plots of bacterial communities. The ordination stress was 0.127. Closed and open symbols indicate DNA and RNA samples, respectively. Correlations between the clustering and frequency of the 10 most abundant OTUs are shown by vectors. Numbers attached to the vectors indicate the abundance rank of the OTUs, which was obtained as follows: the frequency of OTUs was averaged among the replicates in each of the four sample categories (Harding worm/surface and Byron worm/surface), and the sum of these averaged values was used to rank the OTUs. 1. OTU23265 (*Entomoplasmatales*); 2. OTU11203 (“*Ca.* Vermiplasma”); 3. OTU2436 (*Ferruginibacter*); 4. OTU19485 (*Arcicella*); 5. OTU11712 (*Acetobacteraceae*); 6. OTU1618 (*Nakamurella*); 7. OTU15614 (*Methylophilaceae*); 8. OTU8821 (*Rhodanobacter*); 9. OTU742 (*Lachnospiraceae*); 10. OTU6041 (*Rhizobiales*).

Bacterial community structure similarities are depicted by an NMDS plot in Fig. 2. Clear separation among sample categories, except for between DNA and RNA samples, was observed (ANOSIM: global *R*=0.96, *P*<0.001). The contribution of the 10 most abundant OTUs to the separation of the plots is also shown in Fig. 2. “*Ca.* Vermiplasma” OTU11203 greatly contributed to separation between the ice worm and glacier surface samples. *Entomoplasmatales* OTU23265 and *Arcicella* OTU19485 contributed to the grouping of Byron worm samples, and the Harding worm-specific *Lachnospiraceae* OTU742 correlated with the grouping of Harding worm samples. *Ferruginibacter* OTU2436, *Nakamurella* OTU1618, and *Methylophilaceae* OTU15614, which were detected in all sample categories, but were more abundant in glacier surfaces, drove the grouping of the glacier surface plots (Fig. 2).

### Core microbiota associated with ice worms

**Fig. 3:**
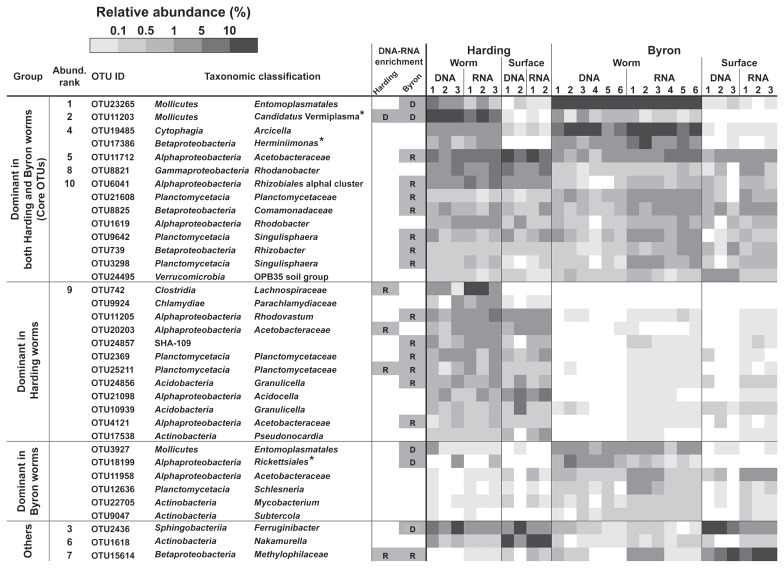
Distribution and abundance patterns of dominant 16S rRNA OTUs associated with ice worms. OTUs showing significant enrichment in either the DNA or RNA samples of ice worms are indicated by “D” (DNA-enriched) and “R” (RNA-enriched). OTU11203, OTU17386, and OTU18199, indicated by asterisks, were classified by our own phylogenetic analysis, whereas other OTUs were classified according to the SILVA database. “Core OTUs”: OTUs accounting for >0.1% of sequences in five or six of the Harding worm samples and also in nine or more of the Byron worm samples (including DNA and RNA samples). “OTUs dominant in Harding/Byron worms”: OTUs found in one of the two glaciers with the above conditions and also in only one or two of the ice worm samples from the other glacier. Blank cells indicate no detection.

In order to identify dominant bacterial OTUs that were consistently associated with ice worms, we first listed OTUs for which the frequency in a sample was >0.1%. These OTUs were then sorted into four groups: (i) OTUs dominant in both Harding and Byron worm samples (designated as “core OTUs”); (ii) OTUs dominant in Harding worm samples only; (iii) OTUs dominant in Byron worm samples only; (iv) other dominant OTUs, which did not fit into groups (i) to (iii), but were included in the 10 most abundant OTUs (see the legend to Fig. 2) (Fig. 3).

Among the core OTUs associated with ice worms, OTU23265 (*Entomoplasmatales*), OTU11203 (“*Ca.* Vermiplasma”), OTU19485 (*Arcicella*), and OTU17386 (*Herminiimonas*) were only rarely found in the glacier surface samples (Fig. 3). *Entomoplasmatales* OTU23265 and “*Ca.* Vermiplasma” OTU11203 phylogenetically belonged to animal-associated bacterial lineages (27), whereas *Arcicella* OTU19585 and *Herminiimonas* OTU17386 were closely related to sequences derived from components of the cryosphere or other freshwater environments (Fig. S2). The other core OTUs were frequently and abundantly detected in both ice worm and glacier surface samples.

The majority of OTUs that were only dominant in either the Harding or Byron worm samples were also abundantly recovered from their corresponding habitat glacier surface samples, but were less abundantly detected in the other glacier surface samples (Fig. 3). However, several OTUs, such as OTU742 (*Lachnospiraceae*), OTU9924 (*Parachlamydiaceae*), OTU3927 (*Entomoplasmatales*), and OTU18119 (*Rickettsiales*), were specifically detected in either the Harding or Byron worm samples, but were almost never found in any of the glacier surface samples. *Parachlamydiaceae* OTU9924 showed the highest sequence similarity (92.5%) to “*Candidatus* Metachlamydia lacustris” (GQ221847), which is a parasite of the aquatic amoeba, *Saccamoeba lacustris* (8). OTU18199 belonged to the uncultured *Rickettsiales* clade LWSR-14 in the SILVA v111 database, but shared only 84.3% sequence identity with its closest relative in the database, *Sphingorhabdus marina* (KT899836) isolated from Ny-Alesund, Svalbard. OTU3927 was closest to the *Entomoplasmatales* core OTU, OTU23265.

### Specificity of bacteria associated with ice worms

As described above, dominant OTUs in ice worm samples included ice worm-specific and glacier-indigenous lineages. In addition to these dominant OTUs, several less abundant OTUs, such as three *Rickettsiales* OTUs (OTU21806, OTU13604, and OTU21100), were almost exclusively detected in ice worm samples (Fig. S3). OTU21806 was closely related to the “*Candidatus* Megaira polyxenophila” clade, which includes endosymbionts of aquatic eukaryotic microbes (31). OTU21806 shared 99.8% sequence identity with endosymbionts of the unicellular green alga, *Carteria cerasiformis* (AB688628) (21), and of the ciliate, *Diophrys oligothrix* strain DS12/4 (FR823003) (31). OTU13604 and OTU21100 showed only low sequence similarities to database sequences: 92.6% with a clone obtained from earthworm nephridia (JX644354) and 84.8% with a clone obtained from a termite gut (AB522149), respectively.

OTU23058 (*Flavobacterium*) was also specifically detected in the ice worm samples, particularly in Byron worms (Fig. S3). This OTU had an identical sequence with clone Ms-09-St2w-2-042 (AB990379) that was previously obtained from ice worms (27). This sequence was the closest (95.7% identity) to the sequence of the isolate, *Flavobacterium* sp. SIB A4(8) (DQ628949), which was obtained from a Canadian glacier; this *Flavobacterium* OTU likely originated in the glacier, as we suggested for the *Arcicella* and *Herminiimonas* core OTUs. OTU21396 (*Brevinema*), which was exclusively detected in the Byron worm samples, shared 91.1% sequence identity with clone MspOz-28-Sw-1-012 (AB991225) obtained from *Mesenchytraeus nivalis*, which was collected from snow in Japan (Fig. S3) (27, 42). The second-closest sequence was *Brevinema andersonii* strain CT11616 (NR_104855); however, sequence identity was only 84.2%.

### Differences in taxonomic compositions between DNA and RNA samples

An analysis using DESeq2 indicated that 62 out of 1,114 OTUs and 217 out of 2,878 OTUs in Harding and Byron worm samples, respectively, showed significant differences in their abundance between DNA and RNA samples (Fig. 3). For example, three *Mollicutes* OTUs (OTU23265, OTU11203, and OTU3927) were significantly more abundant in DNA samples than in RNA samples. Conversely, several glacier-indigenous OTUs of *Alphaproteobacteria*, *Betaproteobacteria*, and *Planctomycetacia*, including OTU11712 and OTU20203 (*Acetobacteraceae*), OTU8825 (*Comamonadaceae*), OTU15614 (*Methylophilaceae*), and OTU25211 (*Planctomycetaceae*), were more abundantly detected in RNA samples. The dominant *Lachnospiraceae* OTU742 was also significantly more abundant in RNA samples (Fig. 3).

### *In situ* localization of *Arcicella* OTUs

**Fig. 4:**
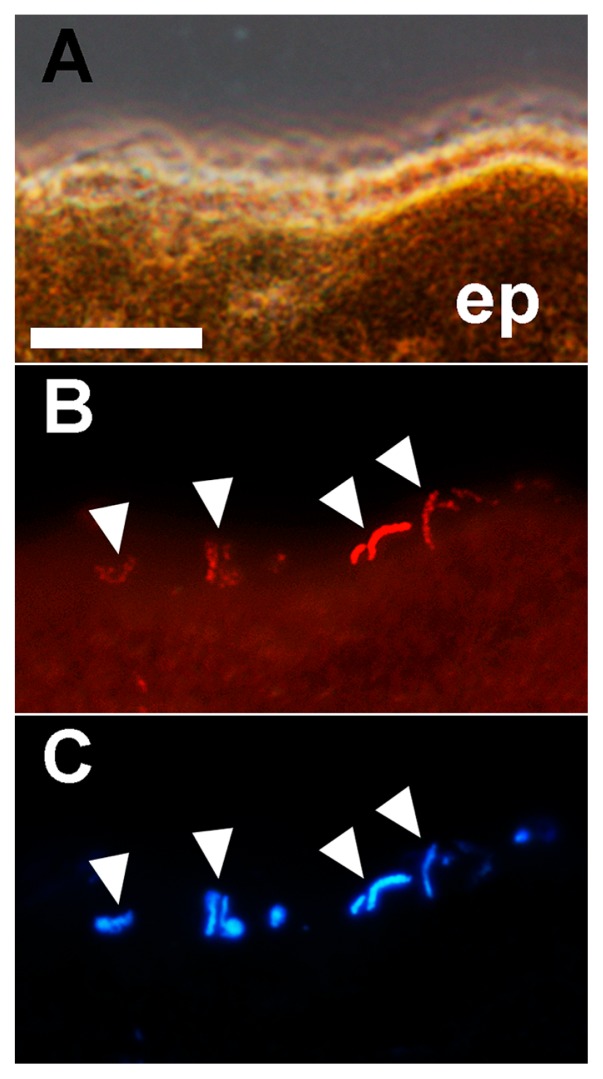
*In situ* detection of *Arcicella* cells in a cross section of an ice worm. An ice worm specimen collected from Byron Glacier is shown as an example. (A) Phase-contrast image. (B) FISH image. Red signals indicate *Arcicella* cells. (C) DAPI-stained image. Arrowheads indicate *Arcicella* cells. ep: epidermis. Bar=10 μm.

We examined the *in situ* localization of *Arcicella* OTU cells, which were phylogenetically affiliated with a glacier-indigenous lineage, but greatly enriched in the ice worm samples (Fig. 3). A FISH analysis revealed that *Arcicella* cells specifically colonized in clusters on the outer surface of the epidermis of Harding and Byron worms (Fig. 4). Approximately 20–50 cells were observed in each 10-μm-thick section of the ice worms. The *Arcicella* cells observed were vibroid, 2–5 μm in length, and resembled other *Arcicella* species described previously (1).

## Discussion

The deep sequencing of 16S rRNA genes and their transcripts clearly revealed specific and facultative relationships between bacteria and glacier ice worms. Significant differences were observed in bacterial community structures between ice worms and their associated glacier surface samples. In particular, several OTUs were almost exclusively detected in the ice worm samples. Among them, the “*Ca.* Vermiplasma” and *Lachnospiraceae* members belong to lineages specific to animal intestinal tracts (27). Although the physiology of “*Ca.* Vermiplasma” currently remains unknown, *Lachnospiraceae* bacteria reportedly participate in polysaccharide fermentation in the gut of terrestrial earthworms (30, 32). Comparative genomics of *Clostridiales* families (*Lachnospiraceae*, *Ruminococcaceae*, and *Clostridiaceae*) also indicated that *Lachnospiraceae* and *Ruminococcaceae* bacteria in the mammalian gut are more specialized for the degradation of plant-derived recalcitrant substrates, such as cellulose, than *Clostridiaceae* bacteria (4). Since algal cells are one of the main food sources of ice worms (17, 27), *Lachnospiraceae* members may play an important role in the digestion of algal polysaccharides in the ice worm gut.

Several *Rickettsiales* and *Parachlamydiaceae* OTUs were also almost exclusively detected in the ice worm samples. These OTUs are phylogenetically related to parasites or endosymbionts of eukaryotes, including algae, ciliates, and amoebas. These OTUs may have been derived from intracellular bacteria that were hosted by ingested eukaryotic organisms, such as green algae and ciliates. However, the frequency of the chloroplast 16S rRNA gene or transcript sequences, which should represent the abundance of food-derived sequences, did not markedly differ between the ice worm and glacier surface samples (Table S2). Therefore, it is more likely that *Rickettsiales* and *Parachlamydiaceae* members specifically inhabit the ice worm body, although the localization of these bacteria remains unknown. Alternatively, these bacteria might proliferate in the ice worm body by infection from ingested eukaryotic organisms; it has been reported that certain members of the *Rickettsiales* or *Parachlamydiaceae* can be horizontally transferred from their original hosts to phylogenetically distinct host organisms by ingestion or mucosal infection (26, 39, 43).

It is notable that *Arcicella* OTU19485 and *Herminiimonas* OTU17386, which belong to glacier-indigenous lineages, were greatly enriched in the ice worm samples. Indeed, the colonization of *Arcicella* cells was observed on the surface of the ice worm body (Fig. 4). These results indicate that glacier-indigenous bacteria can form tight associations with ice worms, and also suggest that these bacteria have been specialized to associate with ice worms. *Arcicella* and *Herminiimonas* are both aerobic and heterotrophic bacteria; the former utilize various carbohydrates (7) and the latter utilize organic acids as carbon sources (15, 20). *Arcicella* species potentially feed on mucus and excrement secreted by the ice worm, and *Herminiimonas* species, although their localization remains unknown, may incorporate metabolites produced by the host or other bacterial species.

Marked differences in OTU compositions between the Harding and Byron ice worm samples were largely attributable to differences in bacterial community structures between the Harding and Byron glacier surfaces (Fig. 2 and 3), which may be caused by environmental differences between these two glaciers. For example, the sampling sites in Harding were around the center of the large icefield, in which any influence from the shore should be minimal, whereas those in Byron were located in a small avalanche cone, which was contiguous with soil and covered with plant fragments possibly derived from shore vegetation. These locational differences may lead to different physicochemical and nutritional conditions, which have a marked impact on the bacterial microbiota, as reported in previous studies (25, 36). This is analogous to findings obtained in terrestrial earthworms; the bacterial microbiota in the earthworm gut changes based on the soil used as food (41). In addition, several OTUs specific to the ice worm, such as *Mollicutes* OTU23265 and OTU11203, *Lachnospiraceae* OTU742, *Parachlamydiaceae* OTU9924, and *Brevinema* OTU21396, also exhibited differential compositional patterns between the Harding and Byron worms (Fig. 1, 3, and Fig. S3). This might reflect the isolation of ice worm populations. Due to the harsh survival conditions of the ice worm, its populations have been geographically isolated among habitat glaciers (9, 10). Thus, bacterial lineages that specifically inhabit the ice worm body might also be isolated and exhibit different taxonomic compositions in the respective glaciers.

An analysis of the 16S rRNA transcript in addition to the gene sequence can provide information on active bacterial species (5, 36). Interestingly, dominant OTUs in the RNA samples of the ice worms were affiliated with both glacier-indigenous and ice-worm-specific bacterial lineages (Fig. 3 and Fig. S2). The glacier-indigenous OTUs of *Alphaproteobacteria*, *Betaproteobacteria*, and *Planctomycetacia*, and animal gut-specific dominant *Lachnospiraceae* OTUs were significantly (*P*<0.05) more abundant in RNA samples than in DNA samples (Fig. 3); these lineages are candidates of the major active taxa in the bacterial consortium associated with ice worms. This result indicates that some of the glacier-indigenous bacteria are exploited as facultative gut bacteria, as observed in the close associations between terrestrial earthworms and soil-derived facultative gut bacteria (11). Much OTUs in RNA samples (Table S1) may be attributable to the higher activity of minor OTUs, which were concealed behind several predominant OTUs in DNA samples (Fig. S1) (28), although the different preparation steps between DNA and RNA templates such as purification and reverse transcription steps may also affect the observed number of OTUs.

## Conclusion

The results of the present study strongly suggest that not only animal-associated bacterial lineages, such as *Mollicutes*, *Rickettsiales*, *Parachlamydiaceae*, and *Lachnospiraceae*, but also glacier-indigenous bacteria, including *Arcicella* and *Herminiimonas*, benefit from the presence of the ice worm. The body surface and intestinal tract of the ice worm appear to provide a unique habitat, which is potentially rich with nutrients, to microbes in glacier ecosystems with limited resources, thereby driving the formation of a worm-associated bacterial microbiota that is distinct from that in glaciers. Since ice worms are the predominant metazoans in Alaskan maritime glaciers, the relationships between ice worms and bacteria potentially have an impact on the geochemical cycles in glaciers, as reported previously for terrestrial earthworms (11). Future analyses on the metabolism and genomes of ice worms and their associated microbiota will provide a deeper understanding of their ecological functions in glacier ecosystems.

## Supplementary Information
