# Supplementary material for: Bacterial Microbiota Associated with the Glacier Ice Worm Is Dominated by Both Worm-Specific and Glacier-Derived Facultative Lineages
Source: Microbes Environ. 2017 Mar 9;32(1):32–9. doi: 10.1264/jsme2.ME16158 (PMC5371072; doi:10.1264/jsme2.ME16158)
Supplement: Supplementary file 1 [file 32_32_s1.pdf]

Table S1. Samples analyzed in this study.

Sample		Sample status	Template	Read count	Number of OTUs	Chao1 richness	Reciprocal Simpson
Harding worm	1	Snowpack, RNAlater	DNA	108,215	98	113	7.37
			RNA	145,388	1,041	1,168	17.37
	2	Snowpack, RNAlater	DNA	120,546	58	58	2.73
			RNA	22,110	290	484	8.15
	3	Snowpack, RNAlater	DNA	132,829	81	86	5.35
			RNA	167,533	307	413	19.95
Byron worm	1	Ice surface, RNAlater	DNA	78,189	145	159	3.36
			RNA	63,214	2,004	2,563	15.73
	2	Ice surface, RNAlater	DNA	87,226	110	115	4.80
			RNA	57,615	1,649	2,200	11.21
	3	Ice surface, RNAlater	DNA	72,882	168	182	2.44
			RNA	33,162	1,290	2,092	9.65
	4	Ice surface, reared	DNA	86,706	42	45	1.76
			RNA	37,952	487	902	3.35
	5	Ice surface, reared	DNA	74,838	79	91	2.15
			RNA	21,646	524	934	9.10
	6	Ice surface, reared	DNA	70,360	64	64	1.91
			RNA	36,484	677	1,200	6.11
Harding surface	1	Ice surface, RNAlater	DNA	136,447	389	580	16.00
			RNA	38,389	1,053	1,219	12.74
	2	Snowpack, RNAlater	DNA	28,137	70	70	15.70
			RNA	39,885	460	701	12.42
Byron surface	1	Ice surface, RNAlater	DNA	64,490	105	112	11.32
			RNA	54,232	1,186	1,487	41.65
	2	Ice surface, RNAlater	DNA	57,860	158	184	13.33
			RNA	31,694	543	630	28.14
	3	Ice surface, RNAlater	DNA	56,673	86	131	21.93
			RNA	40,403	700	765	19.48

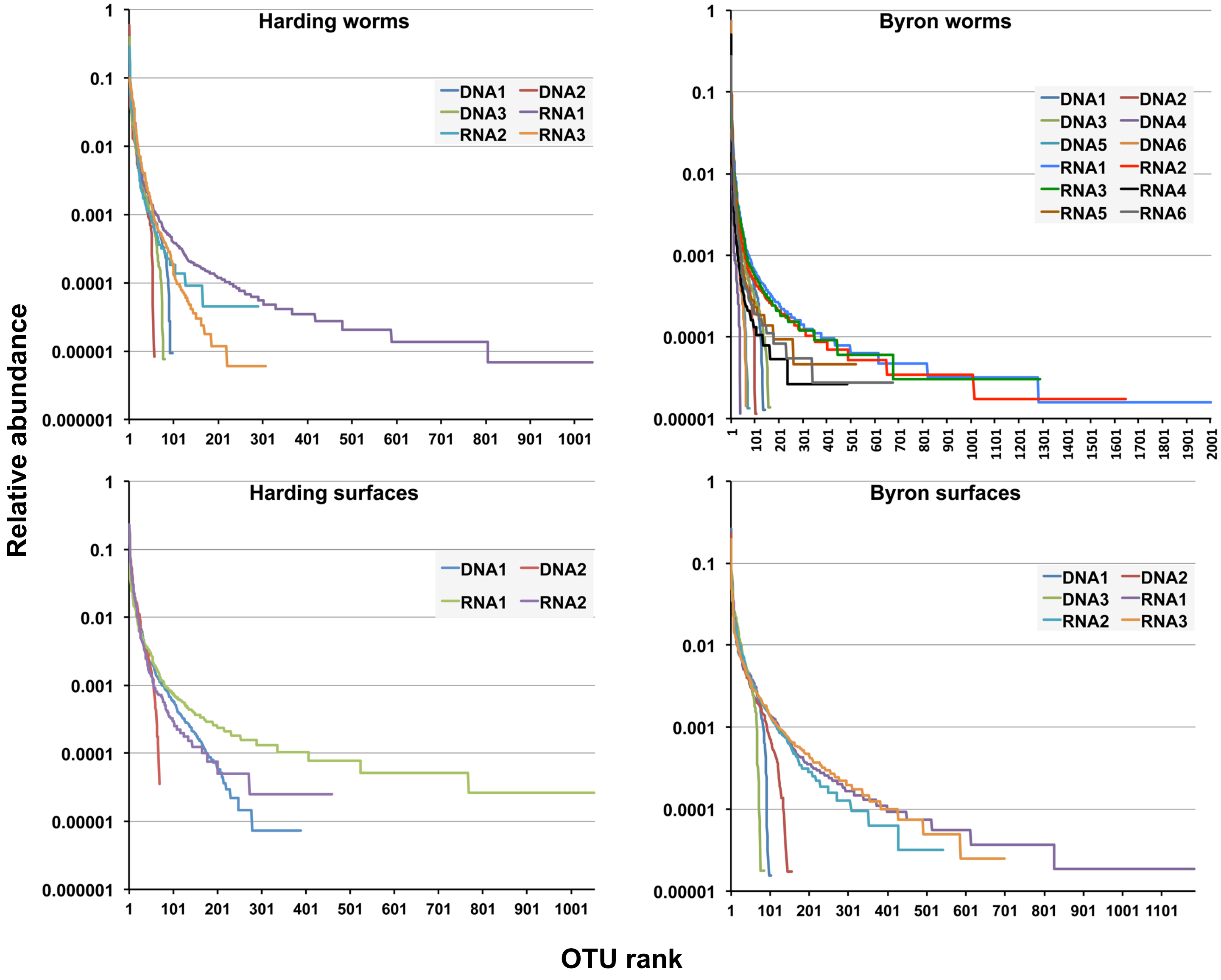

Fig. S1. Rank abundance curves of OTUs in each samples.

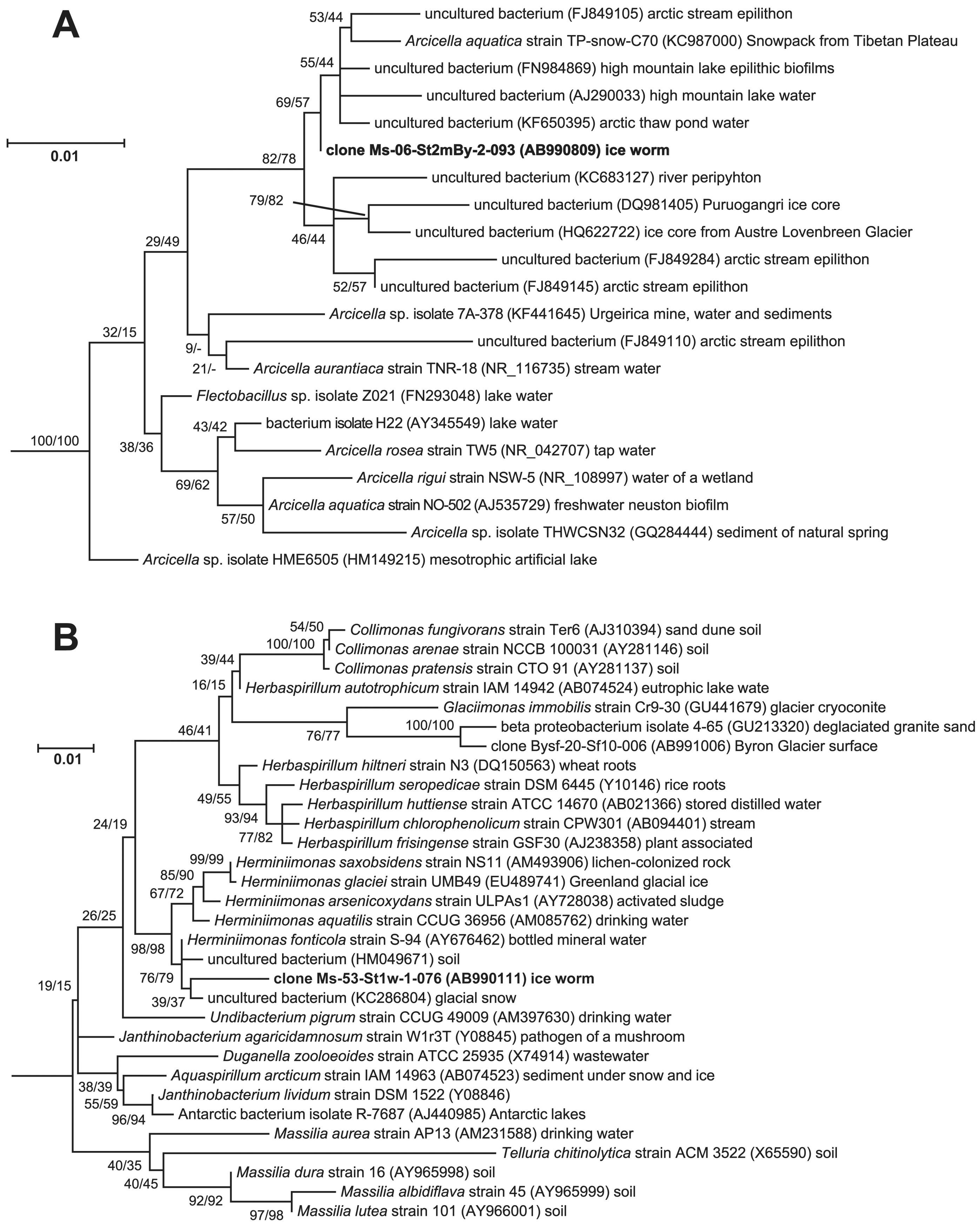

Fig. S2. Phylogenetic positions of dominant *Arcicella* and *Herminiimonas* OTUs specifically detected in ice worm samples. ML tree were constructed based on 16S rRNA gene sequences. Numbers indicate bootstrap support values (ML: left, NJ: right). (A) A phylogenetic tree of *Arcicella* species. “clone MS-06-St2mBy-2-093” corresponds to *Arcicella* OTU19485. *Flectobacillus lacus* strain CL-GP79 (DQ112352) and *Flectobacillus* sp. strain GFA-11 (EU420062) were used as outgroups. (B) A phylogenetic tree of *Oxalobacteraceae* species. “clone MS-53-St1w-076” corresponds to *Herminiimonas* OTU17386. *Burkholderia cepacia* strain ATCC 25416 (AF097530) was used as an outgroup.

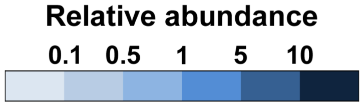

Group	OTU ID	Taxonomic classification		Closest relatives from Murakami et al. (2015): Accession number	Identity (%)	Source
Both	OTU23265	*Mollicutes*	*Entomoplasmatales*	AB990329	100	MS
	OTU19485	*Cytophagia*	*Arcicella*	AB990809	100	MS
	OTU11203	*Mollicutes*	*Candidatus Vermiplasma*	AB989942	100	MS
	OTU17386	*Betaproteobacteria*	*Herminiimonas*	AB990111	99.1	MS
	OTU21806	*Alphaproteobacteria*	*Rickettsiales*	AB991417	92.9	MN
Harding	OTU742	*Clostridia*	*Lachnospiraceae*	–	–	–
	OTU13604	*Alphaproteobacteria*	*Rickettsiales*	–	–	–
	OTU21100	*Alphaproteobacteria*	*Rickettsiales*	–	–	–
	OTU9924	*Chlamydiae*	*Parachlamydiaceae*	–	–	–
	OTU8537	*Alphaproteobacteria*	*Phenylobacterium*	AB991112	99.3	BS
	OTU12934	*Gammaproteobacteria*	*Legionella*	–	–	–
	OTU19393	*Betaproteobacteria*	*Comamonadaceae*	AB989868	97.0	MS
	OTU242	*Spartobacteria*	*Chthoniobacterales*	–	–	–
	OTU21058	*Alphaproteobacteria*	*Reyranella*	–	–	–
	OTU849	*Acidobacteria*	*Candidatus* Solibacter	AB990026	94.3	MS
	OTU22276	*Gammaproteobacteria*	*Xanthomonadales*	AB991162	99.5	BS
	OTU2345	*Deltaproteobacteria*	*Myxococcales* P3OB-42	–	–	–
	OTU15832	*Deltaproteobacteria*	*GR-WP33-30*	–	–	–
Byron	OTU3927	*Mollicutes*	*Entomoplasmatales*	AB990474	99.3	MS
	OTU23058	*Flavobacteriia*	*Flavobacterium*	AB990379	100	MS
	OTU18199	*Alphaproteobacteria*	*Rickettsiales*	–	–	–
	OTU21396	*Spirochaetes*	*Brevinema*	AB991225	91.1	MN
	OTU12636	*Planctomycetacia*	*Schlesneria*	AB989936	100	MS
	OTU16577	*Planctomycetacia*	*Planctomycetaceae*	AB990028	96.7	MS
	OTU22705	*Actinobacteria*	*Mycobacterium*	AB991511	90.3	EJ
	OTU6259	*Planctomycetacia*	*Singulisphaera*	AB990019	96.8	MS
	OTU9047	*Actinobacteria*	*Subtercola*	AB991213	96.1	BS
	OTU11193	*Planctomycetacia*	*Planctomycetaceae*	AB990034	96.7	MS

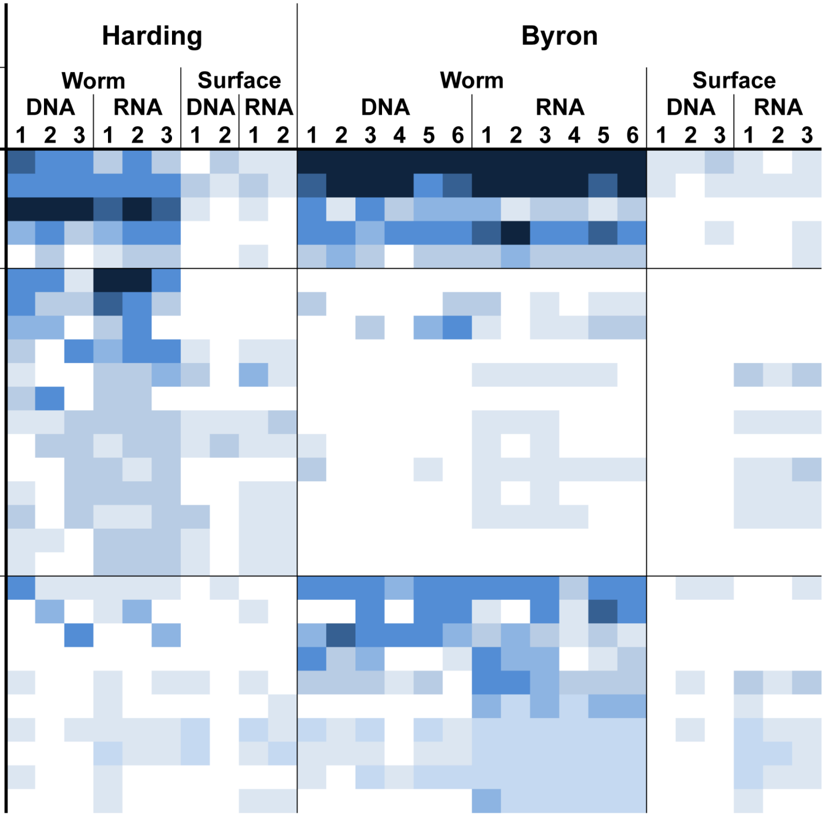

Fig. S3. Bacterial OTUs enriched in the ice worm samples. Among the OTUs that accounted for >0.1% of sequences in any one sample, those found in less than three surface samples (including DNA and RNA samples) in each of the glaciers, were listed. These OTUs were categorized into three groups: "both," which included OTUs that were found in the half or more of ice worm samples from each glacier; "Harding," which included OTUs that were found in more than two Harding worm samples; "Byron," which included OTUs that were found in more than five Byron worm samples. Abbreviations of the clone sources; MS: *Mesenchytraeus solifugus* in Byron Glacier, MN: *Mesenchytraeus nivalis* in Japan, BS: Byron Glacier surface.

Table S2. Read counts and the relative abundance of chloroplast 16S rRNA sequences in each sample

Sample		Template	Number of chloroplast reads
Harding worm	1	DNA	7991 (6.88%)
		RNA	3410 (2.29%)
	2	DNA	5226 (4.16%)
		RNA	432 (1.92%)
	3	DNA	11743 (8.12%)
		RNA	4244 (2.47%)
Byron worm	1	DNA	1011 (1.28%)
		RNA	272 (0.43%)
	2	DNA	277 (0.32%)
		RNA	132 (0.23%)
	3	DNA	238 (0.33%)
		RNA	73 (0.22%)
	4	DNA	213 (0.25%)
		RNA	51 (0.13%)
	5	DNA	902 (1.19%)
		RNA	87 (0.40%)
	6	DNA	171 (0.24%)
		RNA	110 (0.30%)
Harding surface	1	DNA	5627 (3.96%)
		RNA	1980 (4.90%)
	2	DNA	1094 (3.74%)
		RNA	1887 (4.52%)
Byron surface	1	DNA	1986 (2.99%)
		RNA	1148 (2.07%)
	2	DNA	4593 (7.35%)
		RNA	790 (2.43%)
	3	DNA	1568 (2.69%)
		RNA	222 (0.55%)
